# Inhibition of p53-MDM2 binding reduces senescent cell abundance and improves the adaptive responses of skeletal muscle from aged mice

**DOI:** 10.1007/s11357-023-00976-2

**Published:** 2023-10-24

**Authors:** Georgia L. Nolt, Alexander R. Keeble, Yuan Wen, Aubrey C. Strong, Nicholas T. Thomas, Taylor R. Valentino, Camille R. Brightwell, Kevin A. Murach, Sini Patrizia, Harald Weinstabl, Andreas Gollner, John J. McCarthy, Christopher S. Fry, Michael Franti, Antonio Filareto, Charlotte A. Peterson, Cory M. Dungan

**Affiliations:** 1 Department of Physiology, University of Kentucky, Lexington, KY USA; 2 The Center for Muscle Biology, University of Kentucky, Lexington, KY USA; 3 Department of Physical Therapy, University of Kentucky, Lexington, KY USA; 4 Department of Athletic Training and Clinical Nutrition, University of Kentucky, Lexington, KY USA; 5 Department of Health, Human Performance, and Recreation, University of Arkansas, Fayetteville, AR USA; 6 Regenerative Medicine, Boehringer Ingelheim Pharmaceuticals Inc., 900 Ridgebury Road, Ridgefield, CT 06877 USA; 7 Boehringer Ingelheim RCV, Boehringer Ingelheim Pharmaceuticals Inc., Vienna, Austria; 8 Present Address: Department of Health, Human Performance, and Recreation, Baylor University, One Bear Place #97313, Waco, TX 76706 USA

**Keywords:** Skeletal Muscle, Senescence, Senolytics, Regeneration, Hypertrophy

## Abstract

Skeletal muscle adaptation to external stimuli, such as regeneration following injury and hypertrophy in response to resistance exercise, are blunted with advanced age. The accumulation of senescent cells, along with defects in myogenic progenitor cell (MPC) proliferation, have been strongly linked as contributing factors to age-associated impairment in muscle adaptation. p53 plays an integral role in all these processes, as upregulation of p53 causes apoptosis in senescent cells and prevents mitotic catastrophe in MPCs from old mice. The goal of this study was to determine if a novel pharmaceutical agent (BI01), which functions by upregulating p53 through inhibition of binding to MDM2, the primary p53 regulatory protein, improves muscle regeneration and hypertrophy in old mice. BI01 effectively reduced the number of senescent cells *in vitro but* had no effect on MPC survival or proliferation at a comparable dose. Following repeated oral gavage with 2 mg/kg of BI01 (OS) or vehicle (OV), old mice (24 months) underwent unilateral BaCl_2_ injury in the tibialis anterior (TA) muscle, with PBS injections serving as controls. After 7 days, satellite cell number was higher in the TA of OS compared to OV mice, as was the expression of genes involved in ATP production. By 35 days, old mice treated with BI01 displayed reduced senescent cell burden, enhanced regeneration (higher muscle mass and fiber cross-sectional area) and restoration of muscle function relative to OV mice. To examine the impact of 2 mg/kg BI01 on muscle hypertrophy, the plantaris muscle was subjected to 28 days of mechanical overload (MOV) in OS and OV mice. In response to MOV, OS mice had larger plantaris muscles and muscle fibers than OV mice, particularly type 2b + x fibers, associated with reduced senescent cells. Together our data show that BI01 is an effective senolytic agent that may also augment muscle metabolism to enhance muscle regeneration and hypertrophy in old mice.

**Supplementary Information:**

The online version contains supplementary material available at 10.1007/s11357-023-00976-2.

## Introduction  

Skeletal muscle regeneration is an essential biologic process to repair damaged muscle tissue following injury. With aging, muscle regeneration is blunted [1, 2] and is a contributing factor to slower recovery from injury in the elderly. This is due, in part, to muscle from old mice and humans containing fewer, less functional, muscle stem cells, called satellite cells [3–6]. Restoring satellite cells to generate myogenic progenitor cells (MPCs) in old mice improves muscle regeneration [7, 8]. In addition to defects in satellite cell quantity and function, we and others have linked an accumulation of senescent cells following muscle injury to blunted muscle regeneration in old mice [9–11]. Removal of senescent cells using senolytics, such as the cocktail of dasatinib and quercetin (D + Q), augments regeneration, facilitates muscle hypertrophy, and improves physical and muscle function in aged mice [9, 10, 12–14]. Therefore, an intervention that improves MPC proliferation, while simultaneously killing senescent cells, will likely have an additive effect on muscle adaptability with advanced age.

Recent studies have implicated p53 as a therapeutic candidate to restore muscle regeneration in the elderly by modulating both MPC proliferation and senescent cell abundance. Ligand-dependent stimulation of Notch activates p53 and prevents mitotic catastrophe in MPCs isolated from old mice, resulting in augmented MPC proliferation and enhanced skeletal muscle regeneration in old mice [11]. p53 also stimulates ATP production via the upregulation of glycolytic and oxidative pathways [15], which could be valuable for ATP-demanding processes such as muscle regeneration and hypertrophy. Due to its pro-apoptotic function [16, 17], p53 is also a provocative target of senolytic agents. In a mouse model of knee injury, stabilization of p53 by inhibiting the p53-MDM2 interaction using the senolytic agent, UBX0101, induces cell death in senescent cells, leading to improved physical function; however, other muscle-focused outcomes were not measured [18]. In a recent clinical trial in osteoarthritis, UBX0101 failed to reduce pain, suggesting the need to develop new senolytics that target p53.

Although p53 is highly upregulated following a senescence-inducing event, emerging evidence indicates that this is a transient response. Soon after the senescence phenotype has been established, p53 expression is reduced, while negative regulators of p53, such as MDM2, are elevated [19]. p53 promotes cell cycle arrest via p21 [20]; however, p21 remains elevated throughout the induction of senescence, whereas p53 expression and activation declines [19]. In addition to cell cycle regulation, p21 promotes anti-apoptotic pathways to promote cell survival, whereas p53 upregulates apoptotic machinery. The decline in p53 and elevation of p21 is likely a primary mechanism by which senescent cells remain viable, supporting the notion that increasing p53 activity by inhibiting MDM2 with senolytics can be beneficial for clearing senescent cells.

The depletion of MPCs does not lead to muscle atrophy [21, 22] nor accelerate sarcopenia [23, 24]; however, the lack of MPCs does negatively affect muscle adaptation to an external stimulus [22, 24]. Satellite cells isolated from muscle of geriatric mice (28–32 months) appear to undergo senescence *in vitro* more readily than those from adult (5–6 months) or old mice (20–24 months) [25]; however, there is little evidence that satellite cells undergo senescence *in vivo* in resting muscle from old mice [9, 26] or older individuals [3]. Senescent cells are elevated during regeneration [9, 27–30] and hypertrophy [26], and are associated with higher p53 expression and activation, which prevents apoptosis and supports senescent cell survival [19]. The removal of senescent cells with a cocktail of dasatinib and quercetin in old mice facilitates muscle regeneration following injury and increases muscle hypertrophy in response to mechanical overload, with a corresponding increase in MPC abundance [9, 10, 26]. However, dasatinib is a chemotherapy agent that can negatively impact skeletal muscle mass [31, 32], so new effective and safe senolytics are required. In this study, we examined the efficacy of a novel senolytic compound (BI01) that increases p53 stability by inhibiting p53-MDM2 binding, similar to UBX0101, but has a higher affinity for MDM2 and is more stable. Our findings demonstrate that BI01 effectively eliminates senescent cells *in vitro* and *in vivo and* might play a key role in the activation of ATP-generating pathways and contribute to enhanced muscle regeneration and hypertrophy in old mice.

## Results

### BI01 is an effective senolytic agent *in vitro*

**Fig. 1:**
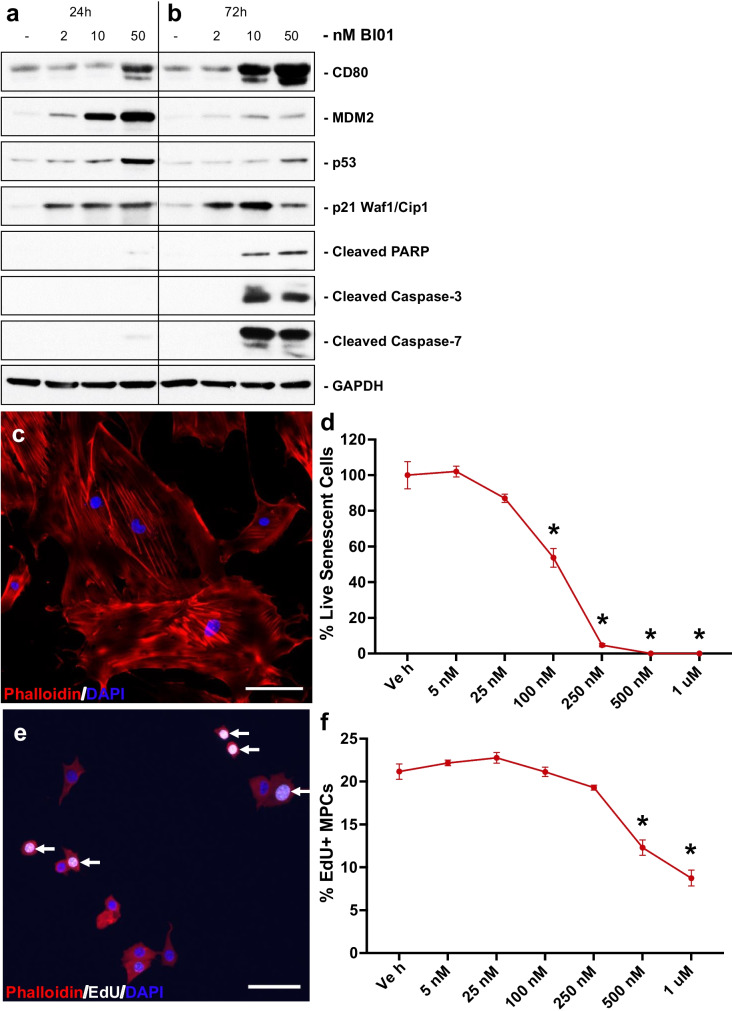
BI01 upregulates p53 expression and kills senescent cells *in vitro*. Protein expression data for SJSA-1 cells treated with increasing concentrations of BI01 for **a)** 24 and **b)** 72 h. **c)** Representative image of mouse primary fibroblasts isolated from old mice induced to become senescent by incubation with H_2_O_2_. **d)** Percentage of live senescent cells after being treated with increasing concentrations of BI01 for 24 h compared to vehicle-treated cells. **e)** Representative image of mouse MPCs isolated from old mice. **f)** Percentage of EdU + MPCs after being treated with increasing concentrations of BI01 for 24 h compared to vehicle-treated cells. Error bars indicate -/ + the standard error of the mean. * p < 0.05 between vehicle and a given concentration of BI01. N = 3 technical replicates, each replicate is an average of n = 5 random images

To assess the ability of BI01 to modulate p53 expression and induce cell death, we performed *in vitro* experiments using SJSA-1 cells, a model of osteosarcoma [33], and freshly isolated mouse primary fibroblasts and MPCs from old mice. Treating SJSA-1 cells with BI01 increased the abundance of both p53 and MDM2, but p53 activity was considerably elevated as shown by higher expression of p53 targets CD80 and p21 and markers of apoptosis (cleaved PARP, cleaved caspase 3, cleaved caspase 9), in a dose-dependent manner (Figs. 1a-b). To determine the effectiveness of BI01 as a senolytic agent, we incubated senescent mouse primary fibroblasts in increasing concentrations of BI01 and quantified the percentage of live cells. We observed a significant reduction in senescent cell abundance at 100 nM BI01 and near complete loss of senescent cells at 250 nM when compared to vehicle-treated cells (Figs. 1c-d). An effective senolytic agent is expected to have little-to-no effect on the proliferation of healthy cells, although induction of p53 has been shown to augment proliferation of MPCs from old mice [11]. Using freshly isolated MPCs from old mice treated with concentrations of BI01 up to the effective dose that killed senescent cells (250 nM), there was no effect on MPC proliferation as assessed by EdU incorporation (Figs. 1e-f), although higher concentrations reduced the percentage of EdU + cells (Fig. 1f). While there were fewer EdU + cells with higher concentrations of BI01, it did not appear to be killing non-senescent cells (Supplemental Fig. 1).

### Administration of BI01 improves muscle mass and function following BaCl_2_ injury

**Fig. 2:**
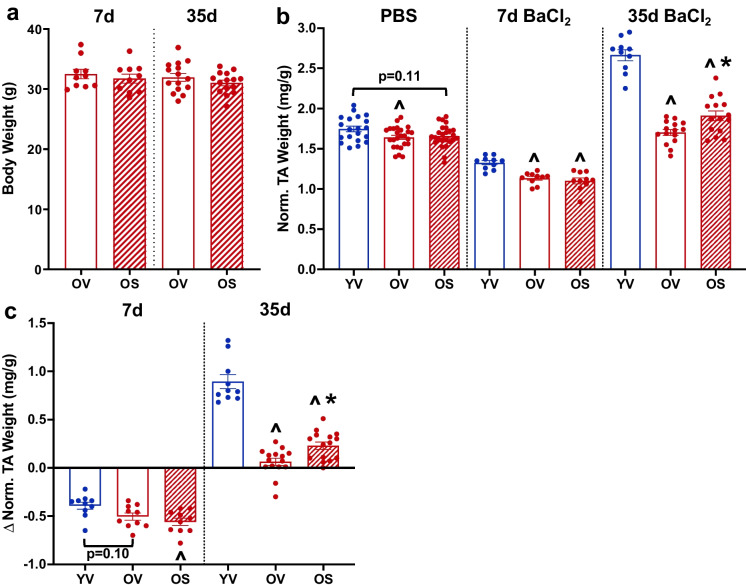
BI01 improves skeletal muscle regeneration in old mice. a) Body weight of young vehicle (YV; blue open bars and blue circle), old vehicle (OV; red open bars and red circles) and old senolytic 2 mg/kg (OS; red hashed bars and red circles) groups 7- and 35-days following BaCl_2_ injury. **b)** Tibialis anterior (TA) weight normalized to body weight following PBS-injection, and 7- and 35-days after BaCl_2_ injection. **c)** Delta normalized TA weight (final minus initial) for YV, OV, and OS mice 7- and 35-days following BaCl_2_ injury. Error bars indicate -/ + the standard error of the mean. ^ p < 0.05 between young and old groups (YV vs. OV; YV vs. OS). * p < 0.05 between OV and OS groups. n = 20–25/group for PBS-injected muscle. n = 10–15/group for 7- and 35-day BaCl_2_-injected muscle

In our previous study, we demonstrated that the senolytic cocktail D + Q effectively eliminated senescent cells and improved muscle regeneration following injury in old mice [9], a finding that has recently been confirmed by others [10]. Using a hit-and-run approach, where mice were treated with BI01 on consecutive days intermittently throughout the study (Supplemental Fig. 2; days denoted on the timeline), we examined the effectiveness of BI01 to improve regeneration in 24-month old mice (OS) 7 days (7d) and 35 days (35d) after BaCl_2_-induced injury to the tibialis anterior (TA) muscle compared to vehicle-treated old mice (OV). The dosage of BI01 used in the animal studies was based on internal pharmacokinetic data provided by Boehringer Ingelheim, which include a C_max_ of 9.2 μm, a t_max_ of 1.7 h, and a mean residence time (MRT) of nearly 6 h (Table 1). Moreover, internal PK and efficacy data from Boehringer Ingelheim show that the minimal effective dose of BI01 was ~ 1.5 mg/kg (AUC_0-24 h_ = 5300 nMh) in an osteosarcoma model (additional pharmacokinetic data are summarized in Table 1). Therefore, we used a dose of 2 mg/kg BI01 for our study. Young adult mice treated with vehicle (YV; 6 months) were used as healthy controls to provide a frame of reference for assessing the impact of BI01 on muscle regeneration in old mice. At both 7d and 35d, BI01 had no effect on body weight in old mice relative to vehicle-treated old mice (compare OS to OV, Fig. 2a). In PBS-injected muscles (pooled from 7 and 35d groups), the normalized TA weight was lower in OV and OS relative to YV, showing no apparent effect of BI01 (Fig. 2b). BaCl_2_ injection reduced normalized TA weight at 7 days, which was unaffected by BI01 (compare 7d OV vs OS, Fig. 2b). The OS group displayed an improvement in normalized TA mass relative to OV at 35d (Fig. 2b), along with having a larger delta in muscle weights (BaCl_2_ minus PBS) relative to OV (Fig. 2c).

**Table 1:**
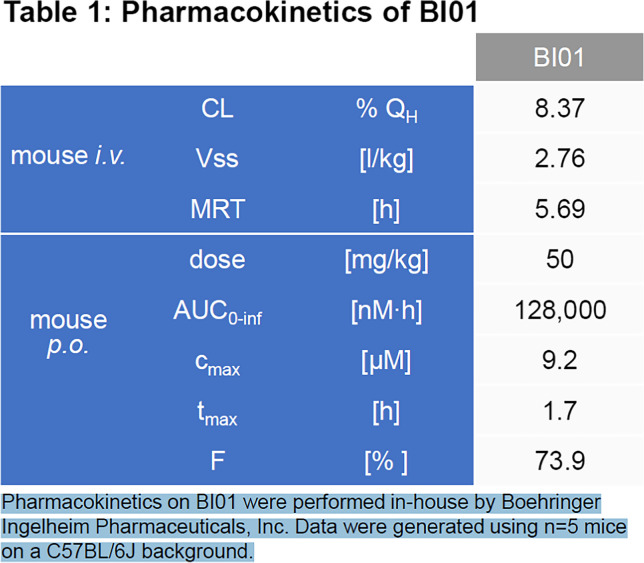
Pharmacokinetics of BI01

Pharmacokinetics on BI01 were performed in-house by Boehringer Ingelheim Pharmaceuticals, Inc. Data were generated using n = 5 mice on a C57BL/6 J background

**Fig. 3:**
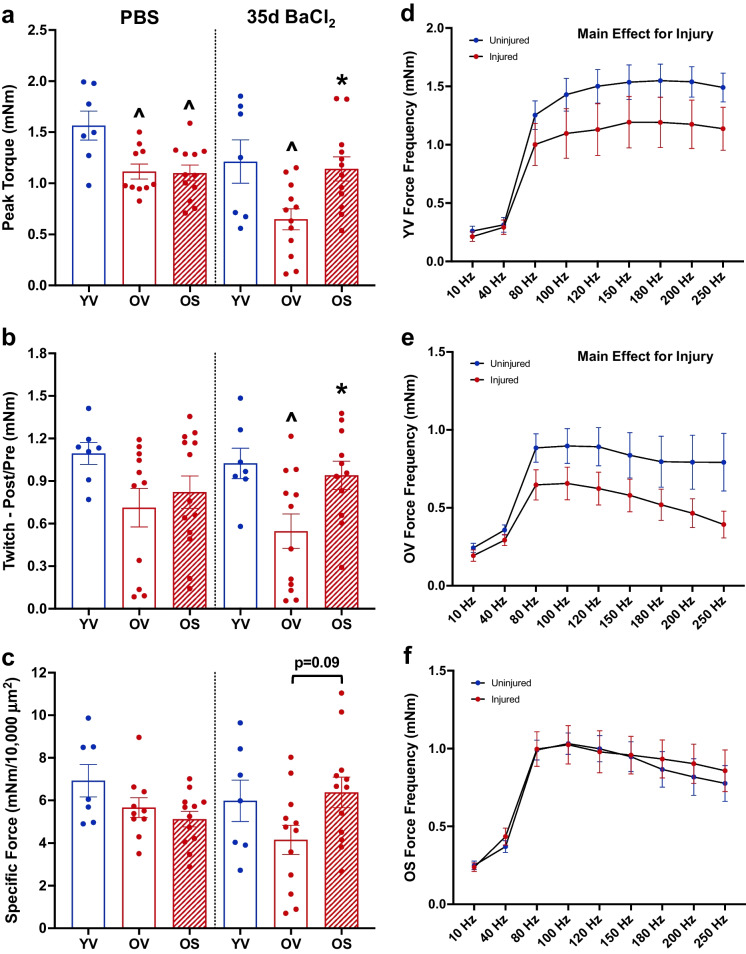
Muscle force and fatiguability is improved following BI01 administration. a) Peak torque from the lower hindlimb dorsiflexors from YV, OV, and OS mice 35-days following PBS or BaCl_2_ injection. **b)** Twitch (peak torque after the force frequency assessment divided by peak torque before the force frequency assessment) from the lower hindlimb dorsiflexors from YV, OV, and OS mice 35-days following PBS or BaCl_2_ injection. **c)** Specific force (peak torque divided by mean muscle fiber cross-sectional area) from the lower hindlimb dorsiflexors from YV, OV, and OS mice 35-days following PBS or BaCl_2_ injection. Force frequency curve for **d)** YV, **e)** OV, and **f)** OS groups. Error bars indicate -/ + the standard error of the mean. ^ p < 0.05 between young and old groups (YV vs. OV; YV vs. OS). * p < 0.05 between OV and OS groups. n = 7–12/group for PBS- and 35-day BaCl_2_-injected muscle

In addition to changes in muscle mass, we also examined the effect of BI01 on *in vivo* muscle function of the dorsiflexors, with the TA being the primary force producer, approximately 35 days following BaCl_2_ injury. BI01 did not affect peak torque, twitch, or specific force in PBS-injected muscles after 35 days (Fig. 3a-c), with both OV and OS groups having lower function than YV. Following BaCl_2_ injury to the TA, YV and OS had a larger peak torque (Fig. 3a) and twitch (Fig. 3b) when compared to OV, and there was a trend for OS to have a higher specific force than OV (Fig. 3c, p = 0.09). Force frequency curves showed a main effect for injury in YV and OV groups, while OS showed similar torque values between injured and non-injured muscles across the force frequency curve (Fig. 3d-f). These results indicate that mice treated with 2 mg/kg of BI01 (OS) had a greater recovery of contractile function following BaCl_2_-injury compared to OV.

### BI01 effectively lowers the senescent cell burden in regenerated muscle *in vivo*

**Fig. 4:**
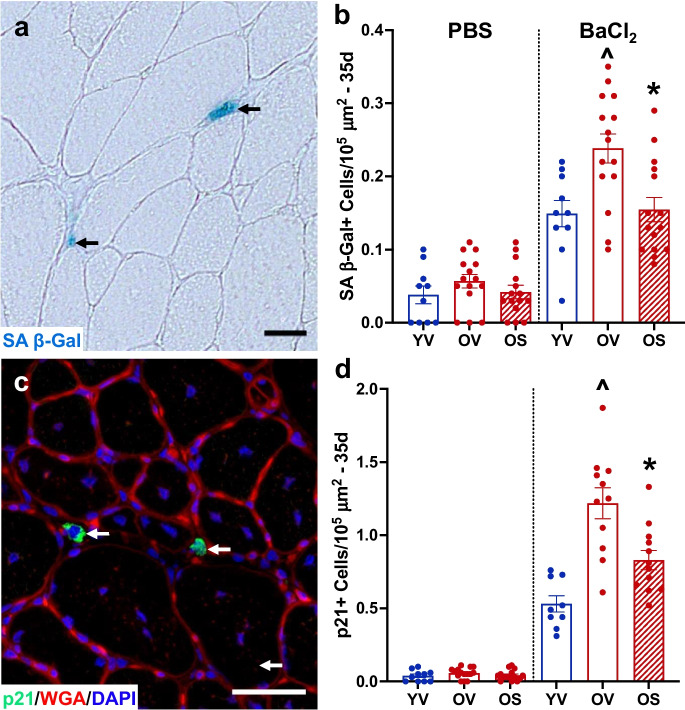
BI01 reduces the senescent cell burden in regenerating muscle. a) Representative image of SA β-Gal staining in the tibialis anterior (TA) muscle (blue region identified with a black arrow). **b)** SA β-Gal + cell abundance per muscle area in YV, OV, and OS mice 35-days following PBS or BaCl_2_ injection. **c)** Representative image of p21 staining in TA muscle (green region identified with a white arrow). **d)** p21 + cell abundance per muscle area in YV, OV, and OS mice 35-days following PBS or BaCl_2_ injection. Error bars indicate -/ + the standard error of the mean. ^ p < 0.05 between young and old groups (YV vs. OV; YV vs. OS). * p < 0.05 between OV and OS groups. n = 10–15/group for PBS- and 35-day BaCl_2_-injected muscle

Using the senescence markers, senescence-associated beta-galactosidase (SA β-Gal) and p21, we quantified the abundance of senescent cells 35 days after TA muscle injury in the old mice. The abundance of senescent cells was not examined at 7 days post injury because 1) the vast majority of the SA β-Gal + cells are macrophages and unaffected by senolytics at this time point following injury [9] and 2) p21 is required for differentiation [34, 35], a process that is highly elevated at this time point. In PBS-injected muscle, there was no difference in SA β-Gal + (Figs. 4a-b) or p21 + (Figs. 4c-d) cell abundance between any group. Thirty-five days following injury by BaCl_2_ injection, senescent cell abundance was significantly higher in OV relative to YV and was reduced by BI01 in muscle (OS, Figs. 4a-d). These data, along with our *in vitro* results, demonstrate BI01 is an effective senolytic agent.

### Muscle fiber cross sectional area (CSA) and satellite cell abundance are positively affected by BI01 following muscle injury

**Fig. 5:**
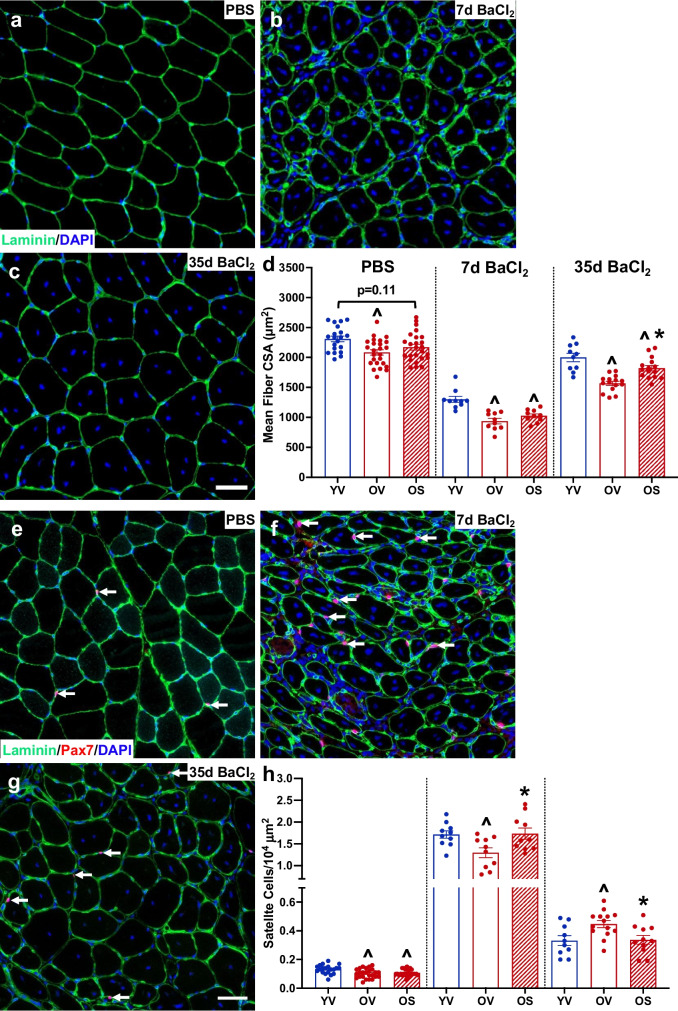
BI01 increases muscle fiber cross sectional area (CSA) 35 days following BaCl_2_ injury. Representative images laminin (green) and DAPI (blue) staining in tibialis anterior (TA) muscle **a)** PBS-injected, **b)** 7 days following BaCl_2_, and **c)** 35 days following BaCl_2_. **d)** Mean muscle fiber CSA values for YV, OV, and OS groups. Representative images laminin (green), Pax7 (red), and DAPI (blue) staining in **e)** PBS-injected, **f)** 7 days following BaCl_2_, and **g)** 35 days following BaCl_2_. **h)** Normalized Pax7 + cells for YV, OV, and OS groups. Error bars indicate -/ + the standard error of the mean. ^ p < 0.05 between young and old groups (YV vs. OV; YV vs. OS). * p < 0.05 between OV and OS groups. n = 20–25/group for PBS-injected muscle. n = 10–15/group for 7-day BaCl_2_- and 35-day BaCl_2_-injected muscle

Consistent with our previous report [9], fiber CSA of control (PBS-injected) TA muscle from OV mice tended to be smaller compared to YV mice; OS fiber CSA was not significantly different from either YV or OV (Figs. 5a,d). Following BaCl_2_-induced injury, fiber CSA was smaller in OV and OS when compared to YV at both 7d and 35d (Figs. 5b-d); however, OS had significantly larger fibers relative to OV after 35 days (Fig. 5d). Given the requirement of satellite cells (Pax7 +) for muscle regeneration [21, 36–39], we examined the abundance of satellite cells in uninjured and injured muscle. In control, PBS-injected muscle, the number of Pax7 + cells was lower in OV and OS mice relative to YV (Fig. 5e,h). Seven days post-injury, OV had fewer satellite cells relative to YV, whereas OS had more satellite cells than OV, not different than YV (Fig. 5f,h). By contrast, at 35 days, OV had more satellite cells than YV and OS (Fig. 5g-h), which likely reflects delayed regeneration in OV mice as reported in our previous study [9].

### Effect of short- versus long-term BI01 exposure on muscle gene expression following injury

**Fig. 6:**
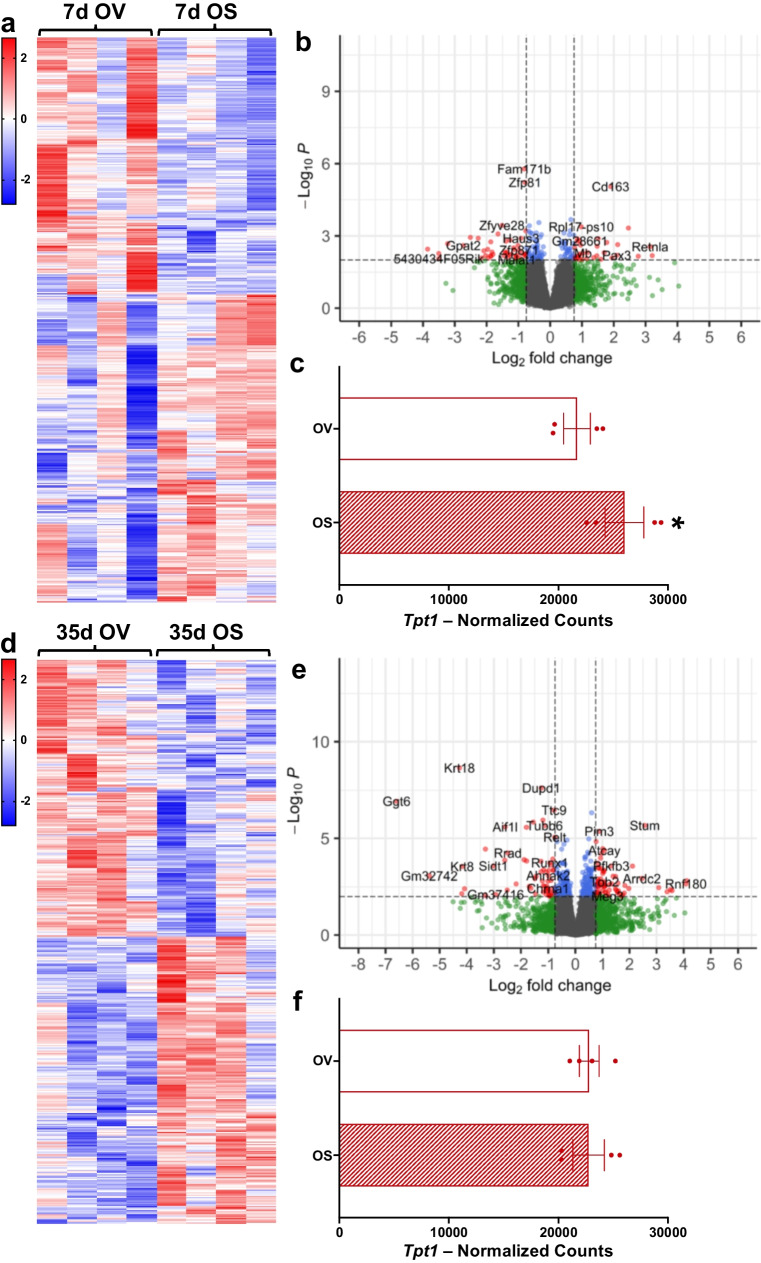
BI01 alters the gene expression profile in 7d and 35d BaCl_2_-injected muscle. **a**) Heatmap of all differentially expressed genes (DEGs) between OV and OS 7 days after BaCl_2_ injury. **b)** Volcano plot of all DEGs between OV and OS 7 days after BaCl_2_ injury. **c)** Normalized counts of *Tpt1* for YV, OV, and OS mice 7-days following BaCl_2_ injury. **d)** Heatmap of all DEGs between OV and OS 35 days after BaCl_2_ injury. **e)** Volcano plot of all DEGs between OV and OS 35 days after BaCl_2_ injury. **f)** Normalized counts of *Tpt1* for YV, OV, and OS mice 35-days following BaCl_2_ injury. Error bars indicate -/ + the standard error of the mean. * p < 0.05 between OV and OS groups. n = 4/group for 7- and 35-day BaCl_2_-injected muscle

Considering p53 impacts numerous genes and pathways, we performed RNA sequencing on TA muscles from OV and OS mice 7- and 35-days after BaCl_2_-induced injury. At both 7d (Figs. 6a-b) and 35d (Figs. 6d-e) post-injury, BI01 treatment impacted the expression of hundreds of genes, leading to the up- and down-regulation of dozens of pathways (Supplemental Figs. 3 and 4). Seven days following injury, relative to OV, OS muscle showed an overall reduction in pathways involving the cell cycle and gene transcription (Supplemental Fig. 3). Pathways involved in translation, ribosome biogenesis, and ATP production (i.e., respiratory electron transport, complex I biogenesis) were all elevated at 7d in OS muscle (Supplemental Fig. 3). Of the genes that were upregulated at 7d in OS muscle, *Tpt1* (TCTP) stands out (Fig. 6c), as it was one of the most abundant genes in our dataset (top 100), is transcriptionally regulated by p53 [40] and can stimulate muscle fiber growth under basal conditions [41]. At 35d post-injury, there were fewer down-regulated pathways in OS muscle than at 7d (Supplemental Figs. 4 and 5) and pathways regulating ATP production were no longer elevated at 35d (Supplemental Fig. 4). The pathways that were downregulated at 7d with BI01 were now elevated at 35d, with pathways involved in gene transcription and the cell cycle being elevated in OS relative to OV (Supplemental Figs. 3 and 4). Further, *Tpt1* was not different between OV and OS by 35d (Fig. 6f). No effect on senescence-associated secretory phenotype (SASP) pathways nor apoptosis were apparent at either time point.

**Fig. 7:**
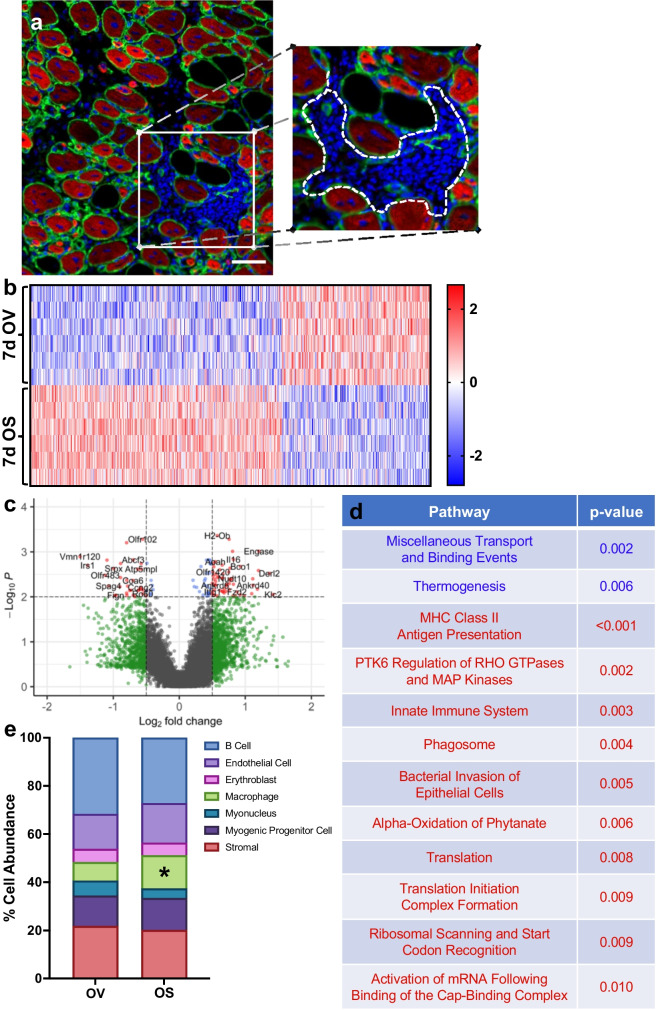
BI01 alters the gene expression profile of non-myofiber nuclei 7 days after BaCl_2_-injury. **a)** Representative image of the regions used (area inside of the white hashed line) for spatial RNA sequencing. **b)** Heatmap of all DEGs in non-myofiber nuclei between OV and OS nuclei 7 days after BaCl_2_ injury. **c)** Volcano plot of all DEGs in non-myofiber nuclei between OV and OS 7 days after BaCl_2_ injury. **d)** Pathway analysis of DEGs between OV and OS groups. **e)** Predictive analysis showing the estimated contribution of various cell populations to the gene expression profile of OV and OS groups. Error bars indicate -/ + the standard error of the mean. * p < 0.05 between OV and OS groups. n = 3/group

mRNA sequencing is a powerful tool to assess transcriptional changes in whole muscle; however, 7 days into regeneration, mononuclear cell populations (i.e., macrophages, fibroblasts, satellite cells) are highly active and greatly increase in abundance. Due to the large contribution of post-mitotic myonuclei, changes in the transcriptome of mononuclear cells can be masked by changes in myonuclear gene expression. Therefore, we utilized spatial RNA sequencing to examine gene expression in all non-muscle fiber nuclei (nuclei outside of the dystrophin-stained sarcolemma) 7 days post injury (Fig. 7a; white dashed line). Similar to whole-muscle mRNA sequencing, there were hundreds of differentially expressed genes between OV and OS (Figs. 7b-c). Consistent with whole muscle sequencing, pathway analysis revealed no effect of BI01 on inflammatory/SASP pathways, but there was upregulation of translation-associated pathways in OS muscle (Fig. 7d). Using the gene expression profile from each sample, we performed a predictive deconvolution analysis to examine theoretical changes in the cellular composition of regenerating muscle with and without BI01. Results from our predictive analysis suggest there is a subtle shift in the overall proportion of each cell type; however, there was a significant elevation in macrophage abundance in BI01-treated mice relative to vehicle (Fig. 7e), which could be beneficial for muscle regeneration, especially during the early stage of the regenerative process.

### BI01 augments gain in muscle mass in response to a growth stimulus

**Fig. 8:**
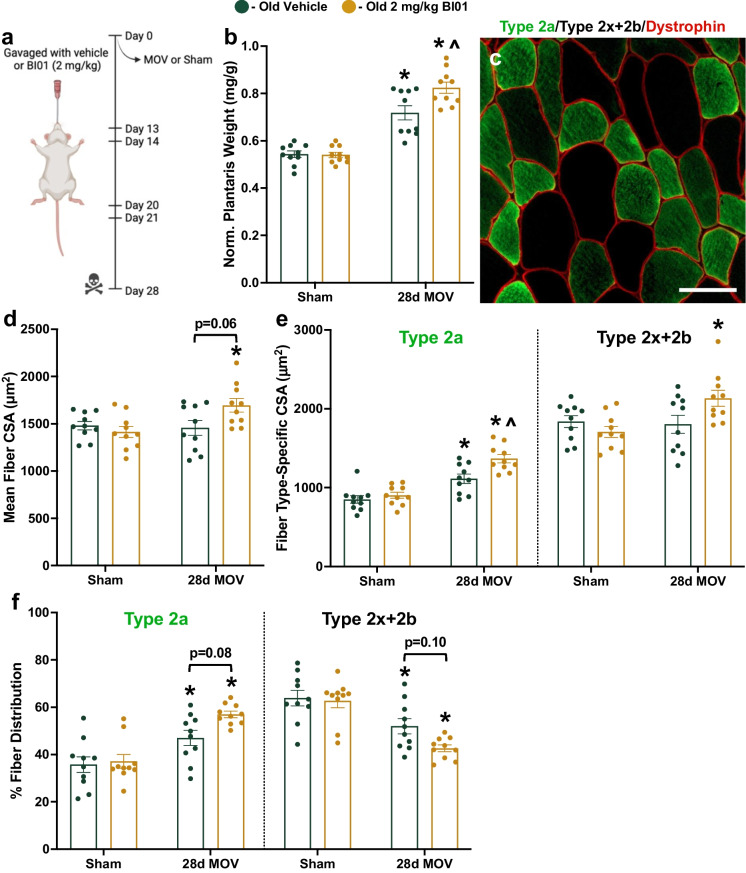
BI01 improves muscle adaptation to mechanical overload (MOV) after 28 days. **a)** Study design schematic. **b)** Normalized muscle mass for sham and 28d MOV groups treated with vehicle (OV; green circles) or BI01 (OS; yellow circles). **c)** Representative image of fiber-type staining for dystrophin (red), Type 2a MyHC (green), and Type 2x + 2b MyHC (black). **d)** Mean fiber CSA for sham and 28d MOV OV and OS mice. **e)** Fiber type-specific CSA for sham and 28d MOV OV and OS mice. **f)** Fiber type distribution for sham and 28d MOV OV and OS mice. Error bars indicate -/ + the standard error of the mean. * p < 0.05 between OV and OS groups. n = 10/group

**Fig. 9:**
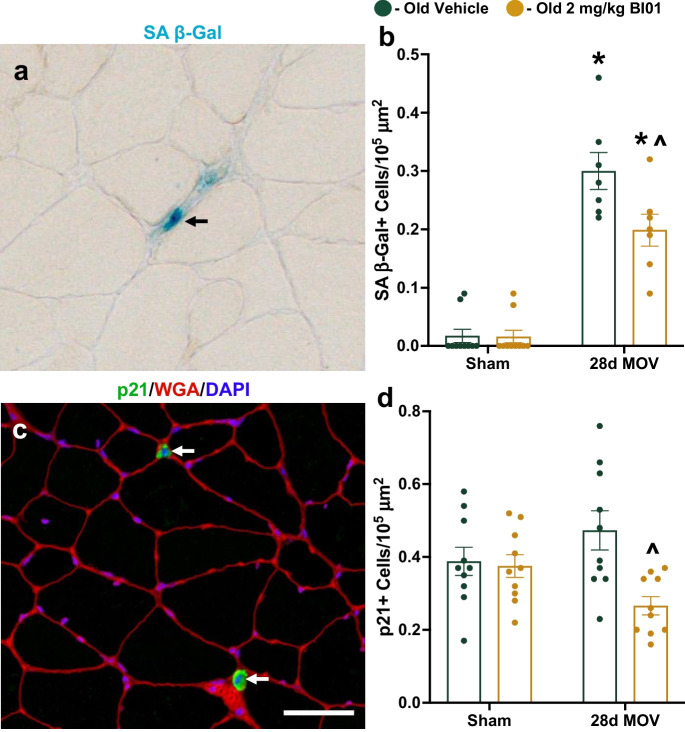
BI01 reduces the senescent cell burden in muscle undergoing hypertrophy. **a)** Representative image of SA β-Gal staining (blue region identified with a black arrow). **b)** SA β-Gal + cell abundance per area for sham and 28d MOV OV and OS mice. **c)** Representative image of p21 staining (green region identified with a white arrow). **d)** p21 + cell abundance per area for sham and 28d MOV OV and OS mice. Error bars indicate -/ + the standard error of the mean. * p < 0.05 between OV and OS groups. n = 10/group

After observing a significant improvement in muscle regeneration, we next wanted to see if BI01 could augment hypertrophic growth in old mice. Mice were pretreated with BI01 and then the plantaris muscle underwent 28 days of mechanical overload (MOV) via synergist ablation surgery with BI101 treatment at days 13, 14, 20, and 21 (Fig. 8a). In response to MOV, both OV and OS groups had larger plantaris muscles compared to their respective sham-operated control; however, muscles from OS mice were larger than OV mice after 28d MOV (Fig. 8b). Only OS had larger plantaris muscle fibers compared to their respective sham control, with a trend for mean fiber CSA to be larger in OS compared to OV (Figs. 8c-d, p = 0.06). Fiber-type specific CSA was also different between groups, as only type 2a fibers were larger in OV mice while both type 2a and type 2x + 2b fibers were larger in OS mice (Fig. 8e). Further, type 2a fibers were larger in OS mice when compared to OV mice (Fig. 8e). There was a shift in plantaris fiber-type distribution towards type 2a fibers in 28d MOV mice compared to sham, and a trend for OS mice to have more type 2a fibers than OV mice (Fig. 8f, p = 0.08). There was a general trend for both OV and OS plantaris muscles to have more myonuclei (Supplemental Figs. 5a-c) and satellite cells (Supplemental Figs. 5d-e) following MOV, with no significant difference between OS and OV. Both OV and OS showed an elevation in SA β-Gal + cells in response to MOV (Figs. 9a-b), while neither group had more p21 + cells (Figs. 9c-d). After being treated with BI01, the OS group had significantly fewer SA β-Gal + and p21 + cells relative to OV (Fig. 9b,d). The elevation of SA β-Gal + cells, without an accompanying increase in p21 + cells, following MOV suggests that a majority of these cells are likely macrophages [9]; however, the reduction in p21 + cells that accompanies the improved response to MOV in old mice treated with BI01 suggests that it may be a viable therapeutic to increase both muscle repair and growth in muscle from older individuals.

## Discussion

Since the original publications showing beneficial effects of senescent cell deletion on lifespan and healthspan [42–44], there has been a large push to develop safe, effective senolytic agents. BI01 is a newly developed MDM2 inhibitor that upregulates p53 and is more stable and with higher affinity than previously described MDM2 inhibitors [18]. We show that *in vitro*, BI01 preferentially kills senescent cells at doses that do not affect non-senescent MPCs. In this study using old mice, we show that a concentration of 2 mg/kg, BI01 effectively improved muscle regeneration following injury resulting in increased muscle size and restoration of contractile function towards a youthful level, as maximal force production and fatiguability were improved in old mice treated with BI01 relative to vehicle. Further, BI01 improved the hypertrophic response in old mice, collectively indicating that BI01 may be an effective senolytic agent to impact multiple age-associated defects in muscle adaptability, following injury and in response to resistance training.

At its core, an effective senotherapy should kill senescent cells and spare healthy ones. Increasing p53 expression by modulating the MDM2-p53 axis is a great target for any new senolytic due to the widespread effects p53 has on cell biology; in particular, the role p53 plays in cell cycle arrest and apoptosis. These effects are largely independent of the mechanism driving p53 expression, as reduction of the MDM2-sparing protein, USP7, disruption of FOXO4-p53 binding, and modulation of MDM2-p53 binding all eliminate senescent cells [18, 45–47], albeit to varying degrees. There appears to be a disconnect between p53 and p21 in senescent cells where p53 decreases and p21 remains elevated [19]. These two proteins have divergent functions, with p53 being pro-apoptotic [48] and p21 exhibiting anti-apoptotic [49] functions in the cytoplasm. Fortunately, BI01 is a powerful p53 activator, and is more effective at killing senescent cells at a low dose than other p53 modulators even in the face high p21 expression. In the present study, BI01 effectively increased p53 and p21 protein expression in cells *in vitro*, in addition to upregulating pro-apoptotic proteins, cleaved caspase-3 and -7 and PARP. This led to cell death in senescent mouse primary fibroblasts, while preserving the proliferative capacity of mouse primary MPCs. These effects were recapitulated *in vivo*, as regenerating muscle from old mice treated with BI01 had fewer senescent cells. In control tissue, BI01 had no impact on satellite cell abundance; however, at 7 days post injury, BI01-treated mice had significantly more satellite cells. This result falls in line with a report by Liu et al., demonstrating greater satellite cell abundance and muscle regeneration in old mice following a pharmacologic increase in p53 [11]. Thus, our results indicate that BI01 is an effective senolytic agent both *in vitro* and *in vivo* by specifically killing senescent cells and appears very effective in improving muscle adaptability in old mice.

p53 has been shown to upregulate the expression of key genes involved in fatty acid oxidation and oxidative phosphorylation [50]. Seven days following TA muscle injury, mice treated with BI01 had higher expression of genes involved in respiratory electron transport and complex I biogenesis, in addition to an upregulation of translational machinery in muscle relative to TA muscles from vehicle-treated mice. On the surface, the simultaneous reduction of transcriptional and cell cycle pathways and elevation of protein translation and ATP-producing pathways by p53 seems paradoxical, Follow-up studies are warranted to further elucidate the therapeutic benefits of elevated p53 expression on energy production as it relates to skeletal muscle adaptation.

Many of the improvements in biological function following the removal of senescent cells have been credited to a reduction in the SASP, as key components of the SASP negatively affect cell homeostasis, including MPC proliferation [51]. In the current study, there was a significant reduction in the muscle senescent cell burden *in vivo* following BI01 treatment, but RNA sequencing showed a limited effect on classic SASP factors; although, we did observe a change in numerous pathways affected by aging. Specifically, there was a reduction in pathways associated with the cell cycle at 7d post-injury, while at 35d post-injury there was a reduction in IL-6 and TNF receptor pathways. Pathways associated with ribosome biogenesis were elevated at 7d, as was gene transcription and IGF- and IRS-mediated signaling at 35d post-injury. While the exact mechanism responsible for the modulation of these pathways is currently unclear, recent work by Englund et al. show an elevation in cell cycle and cytokine receptor pathways, and a reduction in ribosome biogenesis pathways, in skeletal muscle of p21-overexpressing mice [52]. Considering p21 (*Cdkn2a*) expression was lower in BI01-treated mice, p21 could contribute to blunted muscle regeneration during aging.

Mice treated with BI01 had significantly more satellite cells 7d post-injury compared to vehicle-treated mice. Given that macrophages are known satellite cell activators [53] and their secretome is influenced by senolytics [9], we used predictive analysis to determine if BI01 can increase macrophage abundance 7d post-injury. Predictive deconvolution analysis indicated that there was a greater proportion of macrophages in BI01-treated mice, which could explain the differential effects of BI01 on MPC proliferation *in vitro* and *in vivo*. Macrophages are essential for muscle regeneration and release various growth factors and metabolites to promote MPC proliferation and survival, leading to enhanced muscle regeneration and repair [54–56]. Irrespective of MPCs, macrophages are essential for adequate muscle regeneration and hypertrophy, as M1/classically activated macrophages help facilitate the removal of cellular debris and stimulate MPC proliferation [57, 58], whereas M2/alternatively activated phenotype promote MPC differentiation and myotube hypertrophy [58, 59]. In fact, our recent work showed that muscle macrophage content is highly associated with resistance training hypertrophic response in older individuals [60, 61]. p53 delays M1 to M2 polarization [62], which, in the short-term, could be beneficial by expediting the removal of cellular debris and resident senescent cells, in addition to promoting MPC proliferation. Alternatively, BI01 could modulate gene expression in resident macrophages early in the regenerative process to modulate their impact on MPC biology and tissue repair. This is highlighted by our previous work showing > 90% of the SA βgal + cells in skeletal muscle 7 days following BaCl_2_ injury are CD11b + macrophages [9]. In these cells, the gene expression profile was heavily influenced by the D + Q senolytic cocktail, which included an upregulation of *Adamts1* [9], a secreted factor by macrophages to stimulate MPC proliferation [53]. Multiple cellular and molecular mechanisms likely contribute to increased regenerated muscle fiber size in old BI-treated mice, in addition to secreted factors that could facilitate muscle regeneration and recovery [9, 63].

Along with augmenting muscle regeneration, BI01 improved the muscle hypertrophic response to mechanical overload in old mice. This corresponded with BI01-treated mice having larger muscle fibers and fewer senescent cells than vehicle-treated mice in the plantaris muscles in response to MOV. These results extend upon our previous findings that showed improvements in muscle hypertrophy 14 days following MOV in old mice treated with D + Q [26]. Although BI01 effectively reduced the number of senescent cells, there was a stark difference in the number of p21 + cells when compared to the number of SA β-Gal + cells. [9]. Given that the macrophage abundance is heavily upregulated following acute and chronic resistance exercise [64, 65], it is likely that the disconnect between p21 + cells and SA β-Gal + cells is due to the former being truly senescent cells and the latter being mostly CD11b + macrophages9.

Currently, resistance training is the most effective method to increase muscle mass and strength in older individuals, but response is very heterogeneous. Although new senolytic therapies are being developed [66, 67], there is still a need for more therapies to augment muscle growth and regeneration in older individuals. BI01 appears to target multiple processes in muscle that influence muscle adaptability, including senescence, cellular composition (i.e., MPC and macrophage content), and ATP-producing pathways, ultimately increasing muscle size and improving function in old mice. Although BI01 does not appear to affect resting muscle phenotype in old mice, effects in other tissues and lifespan studies are required. Follow-up studies are warranted to expand upon our preclinical data to determine the therapeutic benefit of BI01 on muscle adaptation in older individuals.

## Materials and methods

### Animals

Young (5–6 month; n = 20) and old (24–25 month; n = 140) male C57Bl/6 J mice were purchased from Jackson Labs (Bar Harbor, ME). N = 20 young mice and n = 100 old mice were used for the BaCl_2_ experiments, whereas n = 40 old mice were used for the synergist ablation experiments. All animal procedures were approved by the IACUC of the University of Kentucky. Mice were housed in a temperature and humidity-controlled room, maintained on a 14:10-h light–dark cycle, and food and water were provided ad libitum*.* Mice were euthanized via exsanguination under isoflurane anesthesia, followed by cervical dislocation, and were fasted 6 h prior to euthanization. Following removal of the tibialis anterior (TA), muscles were weighed and ~ 1/3 of the TA was cut lengthwise, flash frozen in liquid nitrogen and stored at -80 °C until total RNA isolation. The other ~ 2/3 of the TA was covered in OCT, frozen in LN_2_-cooled isopentane, and stored at -80 °C for future immunohistochemical analysis.

### *BaCl*_*2*_* injections*

The BaCl_2_ injections were performed as previously described by us [9]. Briefly, the left TA was injected with 1.2% BaCl_2_ (342920, Sigma-Aldrich, St. Louis, MO) in 5 locations equally spaced along the length of the TA muscle with 10 μL of 1.2% BaCl_2_ at each location. The right TA, acting as the control muscle, was injected with phosphate buffered saline (PBS) in the same manner. Mice were euthanized 7- and 35-days following BaCl_2_ injections.

### Synergist ablation surgery

Muscle hypertrophy was induced via the synergist ablation surgical technique, where ~ 1/3 of the gastrocnemius and soleus muscles are removed from the plantar flexor complex, leaving the plantaris solely responsible for plantar flexion (previously described by our lab [26]). Sham controls underwent an identical surgery except for the removal of the gastrocnemius and soleus. Mice were euthanized 28 after surgery.

### Administration of BI01

BI01 is a novel senolytic agent developed by Boehringer Ingelheim Pharmaceuticals, Inc. and was manufactured with > 99.5% purity. BI01 was dissolved in vehicle (0.5% 2-hydroxyethyl cellulose; 434981, Sigma-Aldrich) and administered to the mice via oral gavage using a polypropylene feeding tube (FTP-20–20, Instech, Plymouth Meeting, PA) at a concentration of 2 mg/kg. This dose was chosen based on internal PK and efficacy data from Boehringer Ingelheim that showed the minimal effective dose of BI01 was 1.5 mg/kg (AUC_0-24 h_ = 5300 nMh) in an osteosarcoma model. Pharmacokinetic data are presented in Table 1. BI01 and vehicle were administered using a hit-and-run approached at timepoints defined in Supplemental Fig. 2 and Fig. 8a. To ensure we did not exceed the holding capacity of the average murine stomach, BI01 was dissolved in vehicle at a concentration of 0.06 mg per 100 µL (enough BI01 for a 30 g mouse). Mice were weighed prior to each gavage, with gavage volumes ranging between 80–150 µL depending upon the weight of the mouse.

### *In vivo* muscle function

This protocol was adapted and modified from our previously published methods [68, 69]. Briefly, mice were anesthetized with isoflurane (∼2.5% for maintenance of anesthesia via nose cone; oxygen maintained at ∼1 l/min with a VetEquip vaporizer) and placed on a platform (Aurora Scientific 809c in situ testing apparatus; Aurora, ON, Canada) heated to 37 °C (with an Anova Industries Model 10 water circulator; Stafford, TX, USA) with an attached nose cone for anesthesia maintenance. Fur on both limbs was trimmed (Wahl Bravmini). The leg was braced at the knee via an adjustable clamp with the foot placed in a footplate attached to a dual-mode lever and motor (300D-300C-LRFP, Aurora Scientific). The foot was held static, perpendicular to the tibia, and secured with tape to the force transducer. Platinum needle electrodes were set percutaneously, immediately distal to the knee joint, approximately at the origin of the peroneal nerve. Proper placement was ensured via repeated muscle twitches using the Instant Stim function with Live Data Monitor in Dynamic Muscle Control LabBook (DMC v6.000) An Aurora Physiology system (Model 6650LR Force Transducer, Dual Mode lever System, Hi power Bi-Phase Stimulator, Signal Interface, and software: Dynamic Muscle Control v5.500 and Dynamic muscle Analysis version 5.300) was utilized to determine the proper current for maximal dorsiflexor torque via repeated muscle twitches. The level of electrical current to stimulate maximal torque output was determined by a series of twitches (0.05 s pulse duration) beginning at 1 mA and increasing to approximately 5 mA until the maximum isometric twitch torque stimulated by the minimum current was determined for each mouse. This current remained constant throughout the subsequent torque-frequency curve (10 Hz, 40 Hz, 80 Hz, 100 Hz, 120 Hz, 150 Hz, 180 Hz, 200 Hz, 250 Hz; 0.25 s pulse duration with a 2 min rest period between each stimulus)) to determine peak isometric tetanic torque produced by the dorsiflexor muscles. Both legs were assessed in each mouse, with the PBS limb serving as an internal, healthy control. The frequency that elicited maximal torque output for each mouse was used for rate of torque development calculation. Linear torque-time slope was calculated with a least squares regression fit during twentieth to eightieth percentile of peak tetanic torque.

### Cell culture

Mouse MPCs and fibroblasts were isolated by pooling hindlimb muscles from n = 3 old male mice (24 months) using our previously published protocols [70, 71]. MPCs were cultured in MPC growth media that consisted of Ham’s F-10 Nutrient Mix (11550043, Gibco, Waltham, MA) supplemented with 20% FBS (10082147, Gibco), 1% penicillin–streptomycin (15140122, Gibco), and 5 ng/mL bFGF (100-18B, PeproTech, Cranbury, NJ). Fibroblasts were cultured in fibroblast growth media that consisted of DMEM (11885084, Gibco) supplemented with 10% FBS (10082147, Gibco) and 1% penicillin–streptomycin (15140122, Gibco). SJSA-1 cells were purchased from ATCC and maintained in RPMI-1640 (A1049101, Gibco) supplemented with 10% FBS.

#### Induction of senescence and quantification of live cells

Mouse primary fibroblasts were incubated in 300 μm H_2_O_2_ diluted in fibroblast growth media (described above) for 3 h, washed in sterile PBS, and then cultured for 3 days in fibroblast growth media. Cells were then incubated again in 300 μm H_2_O_2_ diluted in fibroblast growth media for 3 h, washed in sterile PBS, and then cultured for 11 more days in fibroblast growth media. After a total 14 days since the initial H_2_O_2_ insult, an equal number of senescent fibroblasts were plated onto 8-well Nunc™ Lab-Tek™ Chamber Slides (Cat. #177410, Thermo Fisher, Waltham, MA) and incubated in BI01 at concentrations of 5 nM, 25 nM, 100, nM, 250 nM, 500 nM, and 1 μm for 24 h. Senescent fibroblasts incubated in vehicle served as controls. After 24 h, cells were fixed in 4% paraformaldehyde (PFA) for 5 min, washed in PBS, and then counterstained in AF594 conjugated phalloidin (1:100; A12381, Invitrogen, Carlsbad, CA) for 90 min. At the 75 min timepoint, DAPI (1:10,000 diluted in PBS; D1306, Invitrogen) was added to each well for the remaining 15 min. Cells were then washed in phosphate buffered saline (PBS) and cover slipped in PBS:glycerol (1:1). All experiments were performed in technical triplicate. For each replicate, 5 random 10 × images were taken. Cells remaining on the place were counted and expressed as a percentage of cells originally plated.

#### Proliferation assay

Cell proliferation was assessed via an EdU-incorporation assay as described by us [9]. An equal number of mouse primary MPCs (5 × 10 [4]) were placed onto collagen-coated 8-well Nunc™ Lab-Tek™ Chamber Slides (Cat. #177410, Thermo Fisher) and were allowed to adhere for 24 h. MPC growth media supplemented with 5 µm EdU (E10187, Invitrogen) with or without 5 nM, 25 nM, 100, nM, 250 nM, 500 nM, and 1 μm BI01 for another 24 h. After 24 h, MPC growth media was removed, cells were washed in PBS, then fixed in 4% PFA for 10 min. EdU + events were detected using Click-It chemistry, followed by DAPI counterstaining (1:10,000 diluted in PBS; D1306, Invitrogen) for 15 min. Cells were then washed in PBS and cover slipped in PBS:glycerol (1:1). The number of EdU + cells were expressed relative to the total cell number. All experiments were performed in technical triplicate. For each replicate, 5 random 10 × snaps were taken.

### Immunohistochemistry (IHC)

Skeletal muscle samples frozen in OCT were cut using an HM525NX cryostat (Thermo Fisher) at -24 °C and 8 μm sections were placed on charged slides and dried for one hour. Slides were then used for histochemical analysis (described below) or stored at -80 °C for future experiments.

#### Laminin/DAPI

Sections were incubated in primary antibody against laminin (1:200; L9393, Sigma-Aldrich, St. Louis, MO) diluted in PBS for 90 min at room temperature. Sections were then washed in PBS and incubated in secondary antibody against Rb IgG AF488 (1:200; A-11008, Invitrogen) diluted in PBS for 75 min. Sections were washed, incubated in DAPI (1:10,000; D1306, Invitrogen) for 15 min and cover slipped using PBS and glycerol (1:1).

#### Pax7/Laminin/DAPI

Resident satellite cells were labeled using Pax7 as described by us [72, 73]. Sections were fixed in 4% PFA for 10 min, washed in PBS, incubated in 3% H_2_O_2_ for 10 min, and washed again in PBS. Heat-mediated antigen retrieval was then performed by placing sections in 92 °C 10 mM sodium citrate pH 6.5 for 10 min. Once cooled to room temperature, sections were washed in PBS, blocked in 2% BSA plus M.o.M. (Mouse on Mouse; Vector Labs, Burlingame, CA) for 60 min, washed in PBS, and incubated overnight in primary antibodies against Pax7 (concentrate 1:100; PAX7, Developmental Studies Hybridoma Bank) and laminin (1:100; L9393, Sigma-Aldrich) diluted in 2% BSA. The next day, sections were washed in PBS, incubated in Ms IgG1 Biotin (1:1000; 115–065-205; Jackson ImmunoResearch, West Grove, PA) secondary antibody diluted in 2% BSA for 90 min. Following a PBS wash, sections were incubated in streptavidin horseradish peroxidase (1:500; S-911, Invitrogen) and Rb IgG AF488 (1:100; A-11008, Invitrogen) secondary antibodies diluted in PBS for 75 min. Sections were washed in PBS, incubated in TSA AF594 (1:500; B40957, Invitrogen) diluted in DAPI staining solution (1:10,000, D1306, Invitrogen) for 15 min, washed in PBS, and cover slipped in PBS and glycerol (1:1).

#### p21/WGA/DAPI

Our p21 staining protocol was adapted from our previously publish protocol [26]. Sections were fixed in 4% PFA for 10 min, washed in PBS, and blocked in 2% BSA for 60 min. Sections were then incubated in anti-p21 antibody (1:200; ab10199, Abcam) diluted in 2% BSA for 90 min at room temperature, washed in PBS, then incubated in a secondary cocktail containing Rb IgG AF488 (1:100; A-11008, Invitrogen) and wheat germ agglutinin (WGA) AF594 (1:100; W11262, Thermo Fisher) for 90 min. Sections were then washed in PBS, incubated in DAPI staining solution (1:10,000, D1306, Invitrogen) for 15 min, washed in PBS, and cover slipped in PBS and glycerol (1:1).

#### Type 1/Type 2a/Dystrophin/DAPI

Fiber typing and myonuclear quantification was performed as described by our lab [72, 73]. Briefly, freshly cut sections we incubated in primary antibodies against Type 1 MyHC (concentrate 1:100; BA.D5, DSHB), Type 2a MyHC (concentrate 1:100; SC.71, DSHB), and dystrophin (1:100; ab15277, Abcam) for 90 min at room temperature. Sections were then washed in PBS and incubated in isotype-specific secondary antibodies against Ms IgG2b AF647 (1:200; Invitrogen), Ms IgG1 AF 488 (1:200; Invitrogen), and Rb IgG AF 568 (1:100; Invitrogen) for 90 min. After another set of washes in PBS, sections were incubated in DAPI staining solution (1:10,000, D1306, Invitrogen) for 15 min and cover slipped in PBS and glycerol (1:1).

### Senescence-associated beta-galactosidase (SA β-Gal) – skeletal muscle

SA β-Gal was conducted using a published protocol from our lab [3]. Briefly, the sections were fixed in 0.5% glutaraldehyde for 5 min and washed in PBS. Following the wash, sections were incubated in a freshly made staining solution containing 1 mg/ml X-gal in DMF, 5 mM potassium ferrocyanide, 5 mM potassium ferricyanide, 5 M sodium chloride, 1 M magnesium chloride, and 0.2 M citric acid/Na phosphate buffer pH 6.0 ± 0.05. The sections were incubated in the staining solution for 72 h at 37 °C in a dark hybridization oven with fresh staining solution added every 24 h. Sections were then washed in PBS, fixed in 0.5% glutaraldehyde for 10 min, washed in PBS, and cover slipped using PBS and glycerol (1:1).

### Automated muscle fiber CSA analysis

MyoVision [74] was used to quantify the average myofiber CSA and myonuclear abundance. MyoVision automatically determined the CSA of laminin labeled images, with CSAs below 100 µm^2^ and above 6,000 µm^2^ excluded from the analysis. MyoVision also quantified myonuclear abundance by identifying all DAPI + events inside of the muscle fiber border, which was labeled using dystrophin to avoid counting satellite cell-derived nuclei. For all data analysis, regions of the muscle section that appeared to be folded, damaged during cryosectioning, or not damaged by BaCl_2_ (regions with fibers that do not have a central nucleus) were excluded.

### Protein isolation and western blotting

SJSA-1 cells were seeded 24 h prior to drug treatment. After incubation with 2, 10 and 50 nM BI 908763 for 24 and 72 h, cells were lysed on ice with buffer containing 20 mM Tris, pH 7.5, 150 mM NaCl, 1 mM EDTA, 1 mM EGTA, 1% Triton X-100, freshly supplemented with protease and phosphatase inhibitor (ThermoFisher Scientific). Homogenates were centrifuged at 10,000 × g for 10 min at 4 °C to clear cellular debris from the lysate. Total protein concentration in lysates was determined by Bio-Rad Protein Assay according to the manufacturer’s instructions. Equal amounts of total protein were separated by 4–12% Bis–Tris polyacrylamide gel electrophoresis, then transferred onto polyvinylidene difluoride (PVDF) membranes (Bio-Rad Laboratories) and hybridized to specific primary antibodies against CD80 (Cell Signaling #15416), MDM2 (Cell Signaling #86934), p53 (Santa Cruz #sc-126), p21 (Cell Signaling #2947), cl. PARP (Cell Signaling #5625), cl. Caspase-3 (Cell Signaling #9661), cl. Caspase-7 (Cell Signaling #9491), and GAPDH (Cell Signaling #97166) followed by HRP-conjugated secondary antibodies (Agilent) for subsequent detection by enhanced chemiluminescence (Amersham GE Healthcare).

### RNA isolation and analysis

RNA isolation and analysis was performed as previously described [26]. Briefly, total RNA was isolated using the Qiagen miRNeasy Mini Kit (Cat. #217004, Qiagen, Germantown, MD) according to manufacturer’s instructions. RNA was sent to Novogene Co. (Beijing, China) for library preparation and mRNA sequencing. Bioinformatic analysis was performed using *Partek® Flow®* software, v10.0 (St. Louis, MO). Pre-alignment quality control was completed using the default QA/QC tool. Alignment of sequencing reads to the mouse genome (GRCm39) using the splice-aware program STAR (v2.7.8a). Gene counts were quantified using Partek E/M against transcriptome release 103 and a minimum expression cutoff of 10 counts was used to filter out low expression genes. Differential gene expression was analyzed using DESeq2 (v3.5). Gene set over-representation analysis was performed using Consensus Path DB software [75, 76].

### Spatial RNA sequencing and cell deconvolution

Spatial RNA sequencing was performed by NanoString using their GeoMx platform, which was recently described by Danaher et al. [77]. Briefly, freshly cut tissue sections from OV and OS mice 7 days following BaCl_2_ injury were sent to NanoString, who then fluorescently labeled the muscle sections with laminin (muscle fibers) and DAPI (nuclei) using the protocol described here [77]. Slides were then visualized and images were uploaded to NanoString’s online GeoMx Data Analysis software, where we identified 5 regions of interest per section for sequencing. Each ROI was then sequenced and analyzed using the NanoString nCounter® System. Gene expression was then processed by NanoString using their GeoMx Data Analysis software and then the list of differentially expressed genes was downloaded for gene set over-representation analysis using Consensus Path DB software [75, 76]. Using the same data set, we then performed a cell deconvolution analysis to predict the cellular composition of non-muscle fiber nuclei (nuclei outside of the laminin border). Cell abundances were estimated using the SpatialDecon R library^77^, which performs mixture deconvolution using constrained log-normal regression. The 0.75 quantile-scaled data was used as input.

### Statistics

Due to no statistical difference between 7 and 35d groups, data for PBS-injected (control) muscles 7 and 35 days post-injection were pooled for individual groups (YV, OV, OS). For analyses comparing 3 groups (YV, OV, OS), a one-way ANOVA was performed with significance set at p < 0.05. If significance was detected, a Tukey’s post hoc test was used to identify significant comparisons between YV, OV, and OS. A repeated measures ANOVA was used to determine significance for the force frequency experiments. For the MOV experiment, a two-way ANOVA was used with significance set at p < 0.05. If significance was detected, a Tukey’s post hoc test was used to identify significant comparisons between all groups. Statistics were performed using GraphPad Prism 9 software for Mac (GraphPad Software, San Diego, CA).

### Supplementary Information

Below is the link to the electronic supplementary material.

Supplementary file1 (DOCX 13 KB)

## Data Availability

The data that support the findings of this study are available in the supplementary material of this article. RNA sequencing data has been deposited to NCBI Gene Expression Omnibus under accession number PRJNA1029003.
