# Supplementary material for: Inhibition of p53-MDM2 binding reduces senescent cell abundance and improves the adaptive responses of skeletal muscle from aged mice
Source: GeroScience. 2023 Oct 24;46(2):2153–76. doi: 10.1007/s11357-023-00976-2 (PMC10828311; doi:10.1007/s11357-023-00976-2)
Supplement: Supplementary file 1 — Supplementary file1 (DOCX 13 KB) [file 11357_2023_976_MOESM1_ESM.docx]

**Supplemental Figure Legends**

**Supplemental Figure 1.** **BI01 spares healthy cells but blunts proliferation.** Mouse MPCs isolated from old mice are treated with increasing concentrations of BI01. There is no significant difference in the number of DAPI+/EdU- events between groups. Error bars indicate -/+ the standard error of the mean. * p<0.05 between vehicle and a given concentration of BI01. N=3 technical replicates, each replicate is an average of n=5 random images.

**Supplemental Figure 2. Study design schematic.** Study design schematic that shows the timing of drug administration, BaCl_2_-injury, muscle function assessments, and euthanasia (skull icon).

**Supplemental Figure 3. Pathway analysis of DEGs after 7d BaCl_2_-injury.** List of pathways using down- (left) and up- (right) regulated DEGs from OV and OS mice 7 days after BaCl_2_-injury.

**Supplemental Figure 4. Pathway analysis of DEGs after 35d BaCl_2_-injury.** List of pathways using down- (left) and up- (right) regulated DEGs from OV and OS mice 35 days after BaCl_2_-injury.

**Supplemental Figure 5. BI01 has little effect on myonuclear abundance and Pax7 number after 28d MOV.** **a)** Representative image of myonuclear staining for dystrophin (white) and DAPI (blue). This representative image from the same region as shown in Figure 8c. **b)** Mean myonuclei/fiber for sham and 28d MOV OV and OS mice. **c)** Fiber type-specific myonuclei/fiber for sham and 28d MOV OV and OS mice. **d)** Representative image of myonuclear staining for laminin (green), Pax7 (red), and DAPI (blue). **e)** Normalized Pax7+ cells for sham and 28d MOV OV and OS mice. Error bars indicate -/+ the standard error of the mean. * p<0.05 between OV and OS groups. n=10/group.
